# PANDAS Among the Lake District

**DOI:** 10.1192/bjo.2024.669

**Published:** 2024-08-01

**Authors:** Rehaan Khokar

**Affiliations:** Newcastle University, Newcastle, United Kingdom

## Abstract

**Aims:**

Paediatric Autoimmune Neuropsychiatric Disorders Associated with Streptococcal Infection (PANDAS) is an inflammatory brain disorder characterised by a new onset of obsessive-compulsive disorder, triggered by streptococcal infection likely inducing molecular mimicry of antistreptococcal antibody action within brain tissue. PANDAS is perhaps considered controversial in the field of psychiatry due to debates over the validity of the diagnosis, controversy surrounding aggressive antibiotic and immunomodulatory treatment and limited well-controlled case studies.

**Methods:**

A 13-year-old boy, X, presented with new onset worsening confusion, on a background of autism, to a child psychiatric clinic in the Lake District. During the summer, he developed a fixation with Harry Potter and began to act on confabulating beliefs that his mother is (and always has been) Lord Voldemort. X's behaviour became increasingly violent and aggressive and he now only spoke in ‘parseltongue’, refusing to communicate with anyone in any other way. A new personality change was identified as his usual routine behaviours and fixations had dissipated, such as a decrease in his ritualistic behaviours, a loss of his usual inquisitive nature, and an increased fascination with wearing sunglasses due to beliefs that the sun was poisoned by his mother, ‘Lord Voldemort’. Additionally, X's eating habits had markedly changed and now refused all forms of food. Clinically, X also developed a new and sustained tic and was tremulous in clinics, despite no evidence of focal neurological signs. Due to the relatively acute onset of symptoms, an organic cause was queried, which eventually led to the presumed diagnosis of PANDAS.

**Results:**

Extensive investigations, such as an MRI of the brain, autoantibody testing for anti-AQP4, MOG-Ab, and other serological testing, showed no specific cause could be identified other than evident inflammatory changes in the brain. A ‘three-pronged’ treatment approach was adopted: increased psychotherapeutic intervention, antibiotic treatment and IV immunoglobulin therapy.

**Conclusion:**

This case illustrates the importance of recognition of PANDAS and, more pertinently, an appreciation of the biological aspect of the biopsychosocial approach to psychiatry. From the minimal evidence available, there is a suggestion of a relatively good prognosis for patients with suspected PANDAS when intervened timely; however, repeated infections or a chronic course of illness is more difficult to treat. PANDAS remains a diagnostic challenge and perhaps a mystery, with complicated impacts on not only the patient and their families but also the psychiatrist and wider teams involved in the management of care.

